# Supplementary material for: No Excess of Mortality from Lung Cancer during the COVID-19 Pandemic in an Area at Environmental Risk: Results of an Explorative Analysis
Source: Int J Environ Res Public Health. 2023 Apr 14;20(8):5522. doi: 10.3390/ijerph20085522 (PMC10138515; doi:10.3390/ijerph20085522)
Supplement: Supplementary file 1 [file ijerph-20-05522-s001.zip › ijerph-2199359-supplementary.pdf]

Supplementary material

Table S1. Counts and percentages of the comorbidities associated with death in records where lung cancer was a main cause of death.

Comorbidities		n(%)
**ICD10 code**	**Label**	
C34.9	Malignant neoplasm of bronchus and lung, unspecified	74(48.1)
E14.9	Unspecified diabetes mellitus: Without complications	10(6.5)
I11.9	Hypertensive heart disease without (congestive) heart failure	8(5.2)
I10	Essential (primary) hypertension	7(4.5)
I25.9	Chronic ischaemic heart disease, unspecified	6(3.9)
J44.8	Other specified chronic obstructive pulmonary disease	5(3.2)
J44.9	Chronic obstructive pulmonary disease, unspecified	5(3.2)
I51.9	Heart disease, unspecified	3(1.9)
C61	Malignant neoplasm of prostate	2(1.3)
I25.8	Other forms of chronic ischaemic heart disease	2(1.3)
I48	Atrial fibrillation and flutter	2(1.3)
N18.9	Chronic kidney disease, unspecified	2(1.3)
N19	Unspecified kidney failure	2(1.3)
	Other diseases	26(16.9)
	Total	154

Table S2. Counts and percentages of the comorbidities associated with death in records where lung cancer was a secondary diagnosis.

Comorbidities		n(%)
**ICD10 code**	**Label**	
I10	Essential (primary) hypertension	92(10.5)
E14.9	Unspecified diabetes mellitus: Without complications	81(9.2)
I11.9	Hypertensive heart disease without (congestive) heart failure	70(8)
I25.9	Chronic ischaemic heart disease, unspecified	69(7.8)
J44.9	Chronic obstructive pulmonary disease, unspecified	62(7.1)
J44.8	Other specified chronic obstructive pulmonary disease	25(2.8)
I48.9	Atrial fibrillation and atrial flutter, unspecified	19(2.2)
I48	Atrial fibrillation and flutter	16(1.8)
N18.9	Chronic kidney disease, unspecified	16(1.8)
	Other diseases	401(45.6)
	Total	851

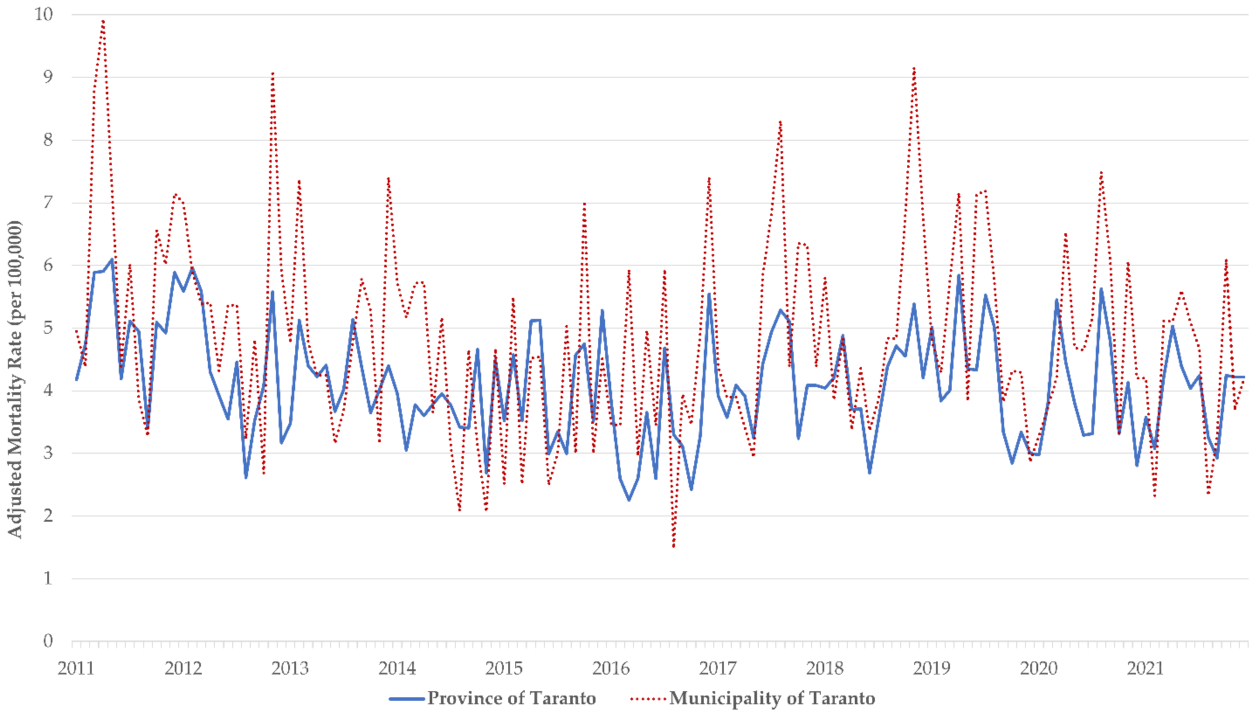

Figure S1. Time series of adjusted mortality rates in the province of Taranto and for the municipality of Taranto in the period 2011-2021.
